# Grape Seed Procyanidins in Pre- and Mild Hypertension: A Registry Study

**DOI:** 10.1155/2013/313142

**Published:** 2013-09-19

**Authors:** Gianni Belcaro, Andrea Ledda, Shu Hu, Maria Rosa Cesarone, Beatrice Feragalli, Mark Dugall

**Affiliations:** Department of Biomedical Sciences, Irvine3 Circulation-Vascular Labs and San Valentino Vascular Screening Project, Gabriele D'Annunzio University, SS 16 Bis 94, Spoltore, Pescara, Italy

## Abstract

The efficacy of a standardized grape seed procyanidins extract (GSPE, Enovita) to decrease blood pressure when associated with nondrug intervention (diet and lifestyle modifications) was investigated in a controlled registry study involving 119 healthy, pre- and mildly hypertensive subjects. Two dosages of Enovita were evaluated (150 and 300 mg/die), using blood pressure and heart rate as the primary endpoints and complementing these observations with a laser Doppler flowmetry (LDF) investigation of the microcirculation state and an evaluation of the plasma oxidative status. After four months of treatment, a statistically significant higher, and dose-dependent, improvement in all endpoints was observed in the treatment groups compared to that of the control, with blood pressure normalizing in 93% of the higher dosage (300 mg) treatment group. Taken together, these observations suggest that GSPEs have beneficial cardiovascular effects that complement current intervention strategies in the hypertension area. The effect on blood pressure adds to the beneficial effects of GSPEs on the cardiovascular disease (CVD) phenotype associated with the oxidation of membrane lipids (endothelial dysfunction, formation of oxidized LDL, and activation of phagocytic cells).

## 1. Introduction

The concept that the nutritional properties of food cannot be recapitulated by, and go substantially beyond, its profile of essential macronutrients (proteins, sugars, and lipids) and micronutrients (vitamins and minerals) is now firmly entrenched in both human and animal nutrition [1]. It explains various once puzzling observations, from the difference between the pharmacological profile of alcohol and wine [2] to the higher susceptibility to parasite infection associated with the nutrition of bees with sugar solutions rather than with honey [3]. Within the differences between alcohol and red wine, one of the most remarkable ones regards the effect on blood pressure [2, 4]. While the regular consumption of alcohol elevates blood pressure of approximately 1 mm Hg for each 10 g of alcohol consumed [2, 4], red wine, at least when associated with moderate drinking (1–4 standard drinks a day), can substantially reverse alcohol-related hypertension and have a beneficial effect on hypertension and overall cardiac morbidity [2]. This effect has been associated with the phenolic constituents of wine, mostly procyanidins (GSPs) [5], and two preliminary intervention studies on prehypertensive patients have supported the view that GSPs lower blood pressure [6, 7]. The mechanist details of this activity have been worked out. Thus, procyanidins directly inhibit angiotensin-converting enzyme (ACE) [8] and indirectly increase the lifetime of endothelially generated nitrogen oxide by inhibiting the production of superoxide, its major physiological scavenger, from endothelial NADP oxidase [9]. The overall result of a decreased peripheral resistance is a reduction of both systolic and diastolic blood pressures, with potential beneficial effects for all risk factors associated with hypertension (stroke, heart disease, congestive heart failure, and kidney disease).

Conventionally, high blood pressure is associated with values of systolic and diastolic pressure higher than 139 mm Hg and 89 mm Hg, respectively, while the corresponding normal values are considered those <120 mm Hg and <80 mm Hg [10]. The grey area between normality and hypertension has been named prehypertension and is generally treated only with diet and lifestyle modifications (self-monitoring, exercise, and relaxation), as often happens for the stage 1 of hypertension, the one associated with 140–159/90–95 mm Hg values [10]. Severe hypertension is an important risk factor for coronary artery disease, more important than high non-HDL cholesterol or obesity [11], but there is mounting evidence that also chronic prehypertension is detrimental for cardiovascular health. Nevertheless, the side-effect profile of hypotensive drugs (diuretics, beta-blockers, and ACE-inhibitors) makes their generalized use in prehypertensive patients questionable [10], providing a rationale to investigate the potential of supplementation with diet-derived agents to promote the attainment of healthy values of blood pressure in this population. In this context, GSPs are the best validated dietary constituents, due to their occurrence in red wine, where contents in the range of 1 g/L are not uncommon, and their identification as the molecular link between wine and its protective cardiovascular properties [5].

GSP is an umbrella name that covers a wide range of products, differing for their contents of monomeric catechins and their degree of oligomerization and decoration with galloyl moieties [12]. GSPs are type B procyanidins, characterized by a single interflavane bond, and represent the best investigated class of condensed tannins [13]. Only the lower homologues (2–5 units) have been shown to be absorbed, either directly or after microbial metabolization. Although the extent of absorption is controversial [14], mass balance experiments with radiolabeled procyanidin B2, a dimeric condensed tannin, have shown an overall significant (>60%) systemic label absorption [15]. For these reasons, we have selected a standardized GSPE (Enovita) enriched in lower oligomers for this study.

## 2. Material and Methods

Enovita, a standardized grape seed extract, and the corresponding blank formulation were provided by Indena (Milan, Italy). Enovita contains ca. 8.6% (HPLC) monomeric procyanidins (catechin, epicatechin, and epicatechin gallate) and ca. 91% OPC (GPC), of which 9% (HPLC) are of the dimeric type. The water content is ca. 5%.

Inclusion criteria for this study were a general good health and borderline hypertension, defined as prehypertension (120–139 mm Hg/80–89 mm Hg) and stage 1 hypertension (140–159 mm Hg/90–99 mm Hg) [10]. The evaluation of general good health involved clinical evaluation and history, full blood test panel to rule out alterations in the lipid profile, fasting glucose or hepatitis markers, an overall normal hematocrit, and proteins profile and coagulation, as well as exclusion of hormonal alterations (thyroid and adrenal).

The nondrug intervention included diet (reduction of salt, alcohol, and caffeinated drinks) and lifestyle (regular exercise, improvement of sleep time, relaxation, and reduction of smoke). No other nutritional elements, vitamins, or drugs were used in the observation period. The same management plan was assigned to all subjects, who were then divided into three groups:

- group 1—300 mg Enovita/day + management plan;
- group 2—150 mg Enovita/day + management plan;
- group 3: controls—management plan only.

Group 1 was made of 37 subjects (14 females), group 2 of 35 subjects (18 females), and group 3 of 47 subjects (19 females) (Table 1). The age range for volunteers was 45–55 years, and the mean age was 51.33 ± 5.31 years in group 1, 49.90 ± 5.31 in group 2, and 49.40 ± 3.00 in group 3. Also, the average BMI was fairly similar within the three groups, namely, 25.41 ± 0.80 Kg/m^2^ in the high dosage group (group 1), 25.20 ± 0.73 Kg/m^2^ in the low dosage group (group 2), and 25.11 ± 0.70 Kg/m^2^ in the control group (group 3) (Table 1). No side effects were reported during the execution of the study, and formulation tolerability (both for Enovita and the corresponding blank formulation) was very good. Based on the number of used capsules, compliance was >94% in group 1 and >95% in group 2. Blood pressure and heart rate were measured digitally. LDF measurements were taken after 30 minutes of supine rest and acclimatization at 21°C for additional 30 minutes. A linear probe (Vasamedics Laserflo, St. Paul, USA) was used for all measurements, taken on the dorsum of foot, and expressed in flux unit [16]. In hypertensive subjects, flux is generally decreased due to vasoconstriction and is increased by treatment, exercise, and control of the risk factors associated with lifestyle.

All measured target parameters (Table 1) have a nonnormal, skewed, or unknown distribution. Therefore, the ANOVA (with the Bonferroni correction) was used to evaluate the before-after results and the Mann-Whitney *U* test for the evaluation of statistically significant differences. A numerosity of at least 20 comparable subjects per group (treatment versus control) was considered necessary to overcome the possible, unavoidable even under the best experimental conditions, differences due to the variability of the microcirculatory target measurements, particularly the laser Doppler test.

## 3. Results and Discussion

GSPs are endowed with high antioxidant activity, orders of magnitude higher than vitamin C, and have been extensively investigated for their capacity to interfere with the development of the cardiovascular disease (CVD) phenotype associated with the oxidation of membrane lipids (endothelial dysfunction, formation of oxidized LDL, and activation of phagocytic cells) [13]. Epidemiological evidence related to the so-called French paradox suggests that GSPs can substantially buffer a high income of animal fats in the diet, although evidence for this activity is controversial because of the difficulty of recapitulating a complex dietary lifestyle in a controlled clinical experiment [5]. On the other hand, there is growing evidence that GSPs can also address another important element of the CVD phenotype, namely, hypertension, and two controlled studies have suggested that the preclinical observations on procyanidins and blood pressure have indeed a clinical translation [6, 7]. These studies were carried out in subjects with avert metabolic syndrome and were of short duration (four weeks). We have now investigated the activity of GSPs in a controlled and longer registry study (four months) on mildly hypertensive but otherwise healthy and only slightly overweight subjects, complementing the observation on blood pressure with a series of other parameters of relevance for the predisease status of the population in study that included heart rate, ECG analysis, and measurements of the microcirculatory status by LDF.

The borderline hypertensive subjects (119) were sorted out in three groups, similar in terms of age and all the objective parameters evaluated, namely, systolic and diastolic blood pressures, microcirculatory status (LDF), heart rate, and plasma oxidative status (Table 2). Group 1 (37 subjects) and group 2 (35 subjects) complemented the management plan with Enovita at two different daily dosages (300 mg/day for group 1 and 150 mg/day for group 2), while in subjects from group 3 (47), only the management plan was implemented and served as the control. The endpoints of the study were evaluated monthly, that is, at weeks 4, 8, and 12, in order to assess the kinetics of development of any beneficial effect.

A decrease of systolic blood pressure was observed in all four groups of the study at month 1, but the decrease was significantly higher in the treatment group (*P* < 0.05). Thus, the average drop of systolic pressure was 28 mm Hg in the high dosage branch and 21 mm Hg in the lower dosage group, while in their respective control groups the decrease was more modest (11 mm Hg). During the next checks, the systolic pressure underwent a further, but much lower, decrease to eventually reach, at the end of the study, an average value of 112 mm Hg in the high dosage group, 123 mm Hg in the lower dosage group, and 141 mm Hg in the control group. In this latter group, no significant further decrease in systolic pressure was observed after the first check. The decrease was, as expected, lower for the diastolic pressure, but a difference between the treatment and the control could still be observed. Thus, in the two treatment groups, a decrease of the diastolic pressure was also observed and developed more gradually in time, eventually reaching 82.3  ±  3.0 mm Hg (from 91.3  ±  2.0 mm Hg) for group 1 and 85.3  ±  2.0 mm Hg (from 91.3 mm Hg) for group 2 (Table 2). Conversely, the decrease was marginal in the control group (from 90.4  ±  2.5 mm Hg to 88.9  ±  3.2 mm Hg). As expected from the data on blood pressure, heart rate was also significantly better reduced in the interventional arms compared to their controls (Table 1), decreasing from 78  ±  3.5 mm Hg to 70  ±  1.5 mm Hg in group 1, from 77  ±  3.4 mm Hg to 72  ±  2.0 mm Hg in group 2, and from 77.2  ±  3.3 mm Hg to 73.2  ±  2.2 mm Hg in the control (group 3).

GSPs have been reported to improve endothelial function and promote microcirculation [16], decreasing the plasma oxidative status [17], and both activities were confirmed in our study (Table 1).

Taken together, our data suggest that GSPs, at least in the profile associated with Enovita, are worth considering to complement dietary and lifestyle changes associated with the attainment of a healthy blood pressure status. The data on blood pressure complement the beneficial activity of GSPs on the CVD phenotype associated with the oxidation of membrane lipids (endothelial dysfunction, formation of oxidized LDL, and activation of phagocytic cells) [17], suggesting that this class of condensed tannins, at least as fractions enriched in lower oligomers, is worth systematic studies for cardiovascular prevention in subjects at risk, providing, for borderline patients, a nondrug option that is better accepted than mainstream medication.

## Figures and Tables

**Table 1:** Key parameters at inclusion, 4, 8, 12, and 16 weeks.

Time	Systolic pressure			Diastolic pressure			Laser Doppler flux			Heart rate			Plasma-free radicals		
	Enovita 300 mg	Enovita 150 mg	Control	Enovita 300 mg	Enovita 150 mg	Control	Enovita 300 mg	Enovita 150 mg	Control	Enovita 300 mg	Enovita 150 mg	Control	Enovita 300 mg	Enovita 150 mg	Control
Inclusion	149; 4.5	150; 3	153.3; 4.4	91; 3.2	91.3; 2	90.4; 2.5	1.1; 0.01	1.1; 0.2	1.1; 0.01	78; 3.5	77.3; 4	77.2; 3.3	378; 22	382; 21	383; 23
4 weeks	121.3; 5.4^∗#^	129; 2.2^∗#^	142.3; 5*	86.3; 3.9*	88; 2*	88.4; 3*	1.28; 0.01^∗#^	1.2; 0.1	1.16; 0.01	73; 2.2*	73; 3.2*	74; 3.2*	—	—	—
8 weeks	119; 4.9^∗#^	125; 3^∗#^	142; 3.9	84.4; 2.8^∗#^	86.1; 2.1*	87; 3.6	2.13; 0.1^∗#^	1.6; 0.2^∗#^	1.22; 0.1	71; 3.1*	73; 2.1	74; 3.1	324; 19^∗#^	339; 32^∗#^	376; 29*
12 weeks	113; 3.3^∗#^	122; 3.2^∗#^	139; 3.5	83; 3.3^#^	86.3; 2^#^	88; 2.2	2.22; 0.2^∗#^	1.9; 0.2^∗#^	1.3; 0.1	69; 2.3*	72.2; 2^#^	74; 2.3	—	—	—
16 weeks	112; 3.5^∗#^	123; 2.1^∗#^	141; 4.3	82.3; 3^#^	85.3; 2^#^	88.9; 3.2	2.23; 0.1^#^	1.9; 0.2^#^	1.2; 0.1	70; 1.5	72; 2.3	73.2; 2.2	317; 22^∗#^	342; 28^#^	374; 22

*(*P* < 0.05) indicates statistical variations in comparison with previous value.

#: better than controls.

**Table 2:** Subjects details.

Treatment	Number of subjects completing the study	Age	Compliance	Tolerability	Dropouts
150 mg + best management	37 (14 females)	51.33; 5.31	94%	Very good	0
300 mg + best management	35 (18 females)	49.9; 5.2	95%	Very good	0
Best management	47 (19 females)	49.4; 3	95%	Very good	3

## References

[1] Hardy G (2000). Nutraceuticals and functional foods: introduction and meaning. *Nutrition*.

[2] Carollo C, Presti RL, Caimi G (2007). Wine, diet, and arterial hypertension. *Angiology*.

[3] Barker RJ, Lehner Y (1973). Acceptance and sustentative values of honey, the sugars of honey, and sucrose fed to caged honey bee workers. *American Bee Journal*.

[4] Puddey IB, Beilin LJ (2006). Alcohol is bad for blood pressure. *Clinical and Experimental Pharmacology and Physiology*.

[5] Corder R, Mullen W, Khan NQ (2006). Oenology: red wine procyanidins and vascular health. *Nature*.

[6] Siva B, Edirisinghe I, Randolph J, Steinberg F, Kappagoda T (2006). Effect of a polyphenoics extracts of Grape Seeds (GSE) on Blood Pressure (BP) in patients with the Metabolic Syndrome (MetS). *The FASEB Journal*.

[7] Sivaprakasapillai B, Edirisinghe I, Randolph J, Steinberg F, Kappagoda T (2009). Effect of grape seed extract on blood pressure in subjects with the metabolic syndrome. *Metabolism*.

[8] Ottaviani JI, Actis-Goretta L, Villordo JJ, Fraga CG (2006). Procyanidin structure defines the extent and specificity of angiotensin I converting enzyme inhibition. *Biochimie*.

[9] Álvarez E, Rodiño-Janeiro BK, Jerez M, Ucieda-Somoza R, Núñez MJ, González-Juanatey JR (2012). Procyanidins from grape pomace are suitable inhibitors of human endothelial NADPH oxidase. *Journal of Cellular Biochemistry*.

[10] Pimenta E, Oparil S (2012). Management of hypertension in the elderly. *Nature Reviews Cardiology*.

[11] Kones R (2010). Rosuvastatin, inflammation, C-reactive protein, JUPITER, and primary prevention of cardiovascular disease—a perspective. *Drug Design, Development and Therapy*.

[12] Quideau S, Deffieux D, Douat-Casassus C, Pouységu L (2011). Plant polyphenols: chemical properties, biological activities, and synthesis. *Angewandte Chemie—International Edition*.

[13] Shi J, Yu J, Pohorly JE, Kakuda Y (2003). Polyphenolics in grape seeds—biochemistry and functionality. *Journal of Medicinal Food*.

[14] Ottaviani JI, Kwik-Uribe C, Keen CL, Schroeter H (2012). Intake of dietary procyanidins does not contribute to the pool of circulating flavanols in humans. *American Journal of Clinical Nutrition*.

[15] Stoupi S, Williamson G, Viton F (2010). In vivo bioavailability, absorption, excretion, and pharmacokinetics of [14C]procyanidin B2 in male rats. *Drug Metabolism and Disposition*.

[16] Salmasi A-M, Belcaro G, Nicolaides AN (1991). Impaired venoarteriolar reflex as a possible cause for nifedipine-induced ankle oedema. *International Journal of Cardiology*.

[17] Maffei Facino R, Carini M, Aldini G, Bombardelli E, Morazzoni P, Morelli R (1994). Free radicals scavenging action and anti-enzyme activities of procyanidines from Vitis vinifera. A mechanism for their capillary protective action. *Arzneimittel-Forschung*.

